# Tissue storage affects lipidome profiling in comparison to *in vivo* microsampling approach

**DOI:** 10.1038/s41598-018-25428-2

**Published:** 2018-05-03

**Authors:** Anna Roszkowska, Miao Yu, Vincent Bessonneau, Leslie Bragg, Mark Servos, Janusz Pawliszyn

**Affiliations:** 1 Department of Chemistry, University of Waterloo, Waterloo, ON Canada; 2 Department of Biology, University of Waterloo, Waterloo, ON Canada; 3 Present Address: Department of Pharmaceutical Chemistry, Medical University of Gdańsk, Gdańsk, Poland

## Abstract

Low-invasive *in vivo* solid-phase microextraction (SPME) was used to investigate the lipid profiles of muscle tissue of living fish. Briefly, mixed mode SPME fibers were inserted into the muscle for 20 min extraction, and then the fibers were desorbed in an optimal mixture of solvents. The obtained lipid profile was then compared and contrasted to that obtained with employment of *ex vivo* SPME and solid-liquid extraction (SLE) from fish muscle tissue belonging to the same group of fish, following a one-year storage period. *Ex vivo* SPME analysis of stored muscle samples revealed 10-fold decrease in the number of detected molecular features in comparison to *in vivo* study. Moreover, i*n vivo* microsampling enabled the identification of different classes of bioactive lipids, including fatty acyls, not present in the lipid profile obtained through *ex vivo* SPME and SLE, suggesting the alterations occurring in the unbound lipid fraction of the system under study during the storage and also indicating the advantage of the *in vivo* extraction approach.

## Introduction

Growing interest in lipidomics over the last few years has led to the discovery and identification of hundreds of novel lipids, revealing their growing importance as it pertains to their involvement in the complex biological transformations taking place in living systems^1,2^. Profound analysis of lipid metabolism has proven that apart from their important roles as structural components of cell membranes and energy reservoirs, lipids are also intermediates of cellular signaling pathways and modulatory ligands for membrane proteins^3–5^. Knowledge concerning their significance in endogenous signaling and metabolism is continuously expanding as the roles of numerous small molecular-weight bioactive lipids are unveiled by lipidomic research, however, the mechanisms of their interaction with other cellular components and their role in tissue stability, is yet to become fully clear^6,7^. Hence, the availability of rapid *in vivo* extraction methods capable of capturing short-lived biological lipids is essential to the unveiling of the highly complex and very dynamic lipid profile^8^.

Currently used protocols for isolation and purification of lipids from biological matrices are tedious and time-consuming. Also, traditional sampling and separation methods used to identify trace lipids are often burdened by limitations related to the characterization of the real composition of living cells^9,10^. For instance, employment of certain steps involved in conventional protocols, such as invasive tissue collection (e.g. biopsy), tissue homogenization, and application of organic solvents during sample preparation, may hinder discrimination between lipids present in free form versus those previously bound to cellular components in the living system, hence introducing errors in the interpretation of the cellular lipidome^11–13^. Moreover, the stability of the lipid profile may also be compromised during sample storage as a result of enzymatic activity (degradation and/or aggregation processes) or chemical alterations^14^.

Aiming to provide adequate sample handling and fast, comprehensive extraction of small molecular weight compounds from tissue, *in vivo* SPME was introduced as a sample preparation method that integrates sampling, extraction, and quenching of metabolites into a single step, preserving the real metabolic profile of the analyzed matrix. *In vivo* SPME does not disturb the biological homeostasis of cells under study due to the solvent-free and non-exhaustive nature of SPME probes^15,16^. Moreover, *in vivo* application of SPME sampling facilitates the capture of low molecular weight and unstable metabolites present in their free form at the cellular level, thus allowing for insights into the intrinsic biochemical pathways and networks of the living system^8,17,18^.

In the present study, we introduced low-invasive SPME fibers with biocompatible coatings to muscle tissue of living fish (*in vivo* SPME) for untargeted lipidomics profiling. In order to analyze the influence of sample storage conditions on the lipidome, including potential alterations to the existing forms of lipids (bound vs. unbound), results from the *in vivo* study were then compared to results obtained from an *ex vivo* SPME study of samples collected from the same group of fish following prolonged storage conditions. Fish muscle samples were stored at −80 °C for a 1 year period, then submitted to *ex vivo* SPME analysis. Both sets of data, obtained from *in vivo* and *ex vivo* SPME, were then compared to results obtained by SLE, performed on homogenized fish muscle samples prepared from the same samples used for *ex vivo* SPME analysis. Annotation of lipids was facilitated by xMSannotator software with the use of a multistep strategy^19^ and was based on intensity profiles, retention time, mass defect, and isotope/adduct patterns of peaks in the data. LIPID MAPS was employed as a reference database for tentative identification of annotated molecules.

## Results and Discussion

In lipidomic studies, the knowledge concerning the sample handling and storage, and their effects on the composition of lipids is limited and not consistent^20,21^. It has been shown that 24 months tissue storage results in compositional changes and decrease of selected lipids^22^, whereas other studies reported increase or decrease of the specific lipid fractions during prolonged storage^23,24^. However, most of the previously reported data are obtained from the experiments performed with the use of exhaustive liquid extraction techniques. The primary purpose of this study was the analysis of lipid metabolites specifically detected using *in vivo* SPME technique and the comparison of annotated features with the results obtained for exactly the same fish samples after one-year storage period using *ex vivo* SPME and SLE in order to examine the influence of storage and handling conditions on the quality of lipid composition.

**Figure 1:**
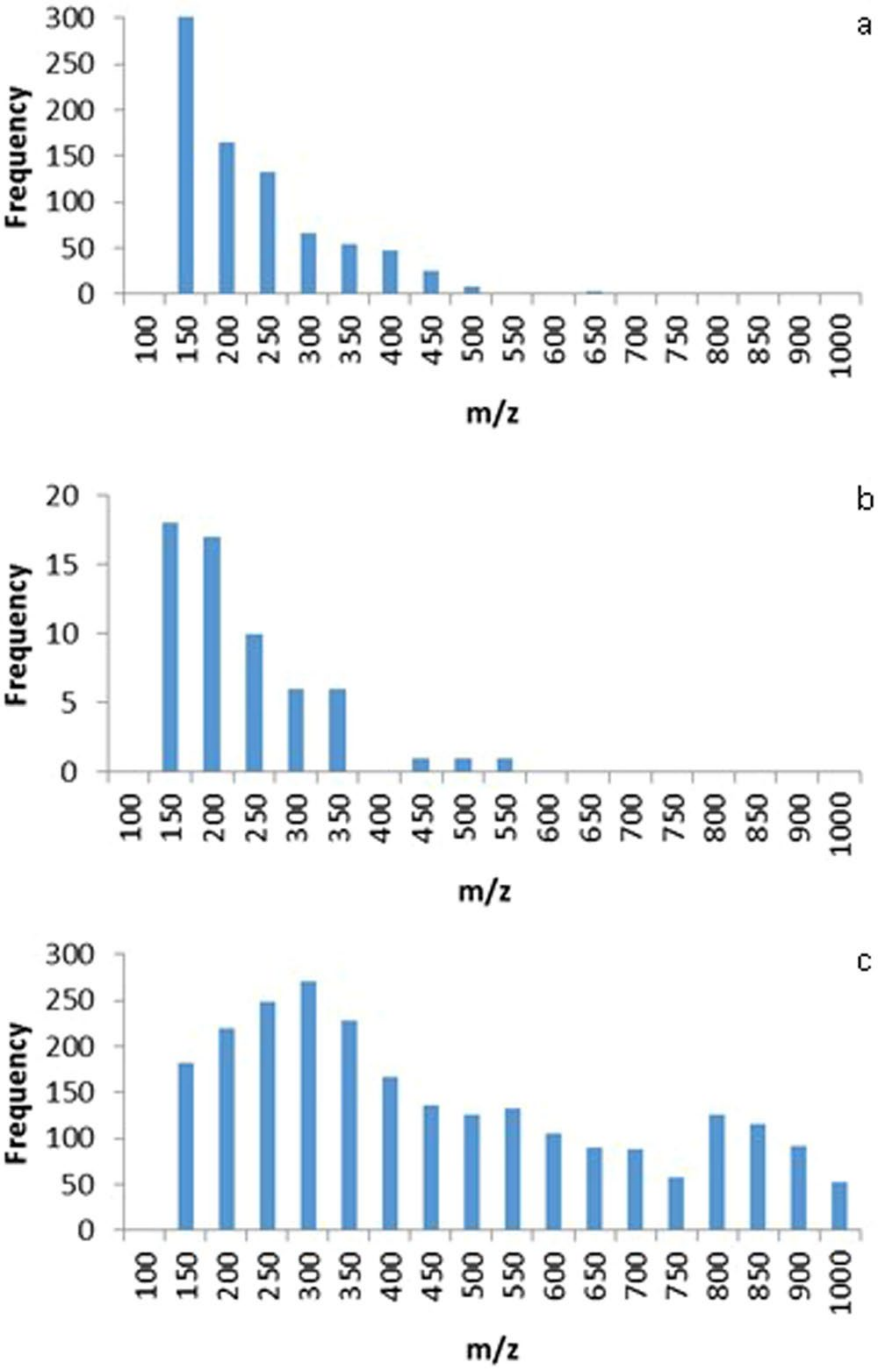
Histogram of mass distributions of detected peaks found in data obtained from (**a**) *in vivo* SPME of fish muscles in living fish; (**b**) *ex vivo* SPME analysis of non-homogenized fish tissue after a one-year storage period; and (**c**) solid-liquid extraction analysis of homogenized fish muscle samples after a one-year storage period.

Characterization of *in vivo* SPME extracts obtained from muscle tissue of juvenile fish revealed that among a total number of 845 detected features with molecular masses ranging from 100 to 500 m*/z* (Fig. 1a), 30% were annotated as lipid species belonging to 3 different lipid categories, namely fatty acyls, sterol lipids and glycerolipids. Although SPME probes enable the extraction of compounds with molecular masses up to 1000 *m/z* that are present in unbound form in the tissue, the majority of the unique lipids annotated with medium to high confidence were low molecular weight fatty acyls (Table 1). Among the annotated lipids, several N-acyl amides, known as the signaling molecules of the living system, were identified in *in vivo* SPME samples and were not found in muscle tissue samples after the one year storage period. Moreover, *in vivo* SPME sampling allowed for the capture of the compound 2-decene-4,6,8-triyn-1-al, annotated as a unique fatty aldehyde, and also the compound 10 hydroxy-8E-decene-2,4,6-triynoic acid, potential end-product of fatty aldehydes conversion to fatty acids, which have been shown to play a pivotal role as a rapid energy source in developing species^25,26^. However, none of these compounds were present in stored fish muscle samples, suggesting the occurrence of lipidome profile alterations during storage (Table 1). Indeed, analysis of stored muscle samples (*ex vivo* SPME) revealed distinct differences in lipid composition, with a 10-fold decrease in the number of detected molecular features in comparison to the number of features observed for *in vivo* SPME samples (Fig. 1b). In addition to the observed decrease in the number of lipids detected *in vivo*, the appearance of a higher molecular mass lipid (above 500 *m/z*), a sterol conjugate - (25 R)-3α,7α-dihydroxy-5β-cholestan-27-oyl taurine, was also observed in *ex vivo* extracts. Comparison between *in vivo* and *ex vivo* SPME was demonstrated as an effective tool in the detection of alterations in the lipidome profile of samples over time, suggesting that unbound compounds detected during *in vivo* SPME of the living system may have undergone binding reactions as a consequence of morphological changes in lipid structure during sample storage.

**Table 1:** Unique features with medium-to-high confidence matches annotated by LIPID MAPS.

Compound	Category	Precursor m/z	Precursor adducts	RT (min)	Sample			Confidence match
					*in vivo* SPME	*ex vivo* SPME	SLE	
2-decene-4,6,8-triyn-1-al	Fatty Acyls	143.04856 145.04431 144.05173	M + H M + 3 H M + 2 H	0.80	✓			high
N-eicosanoyl-ethanolamine	Fatty Acyls	356.35235	M + H	8.10	✓			medium
N-(11Z-eicosaenoyl)-ethanolamine	Fatty Acyls	354.33658	M + H	8.45	✓			medium
4-[(5-aminopentyl)(hydroxy)amino]−4-oxobutanoic acid	Fatty Acyls	219.13410	M + H	0.93	✓			medium
N-octadecanoyl-valine	Fatty Acyls	384.34715	M + H	8.97	✓			medium
N-octadecanoyl-proline	Fatty Acyls	382.33164	M + H	8.60	✓			medium
3-oxobutanamide	Fatty Acyls	102.05523 103.05861	M + H M + 2 H	0.90	✓		✓	medium, high*
hexadecanamide	Fatty Acyls	256.26374	M + H	8.15	✓			medium
pentanamide	Fatty Acyls	102.09158	M + H	1.37	✓		✓	medium
Dodecanamide	Fatty Acyls	200.20115	M + H	6.27	✓		✓	medium
O-(17-carboxyheptadecanoyl)carnitine	Fatty Acyls	458.34762	M + H	7.62	✓			medium
3,7-dimethyl-8,11-dioxo-2E,6E,9E-dodecatrienal	Fatty Acyls	235.13304	M + H	6.43	✓			medium
2-amino-hexanedioic acid	Fatty Acyls	162.07614	M + H	0.93	✓			medium
2E,4E,8E,10E-dodecatetraenedioic acid	Fatty Acyls	223.09658	M + H	6.10	✓			medium
7,8-diaminononanoic acid	Fatty Acyls	189.15971	M + H	1.17	✓			medium
2-amino-3-oxo-hexanedioic acid	Fatty Acyls	176.05533	M + H	0.90	✓			medium
12-amino-dodecanoic acid	Fatty Acyls	216.19599	M + H	4.28	✓			medium
3-oxo-5S-amino-hexanoic acid OK	Fatty Acyls	146.08116	M + H	0.87	✓		✓	medium
10-hydroxy-8E-Decene-2,4,6-triynoic acid	Fatty Acyls	177.05468	M + H	6.10	✓			medium
5,7,9,11,13-tetradecapentaenoic acid	Fatty Acyls	219.13809	M + H	5.95	✓			medium
octadecanamide	Fatty Acyls	284.29440 286.30212	M + H M + 3 H	8.85	✓		✓	medium, high*
N-(9Z,12Z-octadecadienoyl)-ethanolamine	Fatty Acyls	324.28921 325.29257	M + H M + 2 H	7.98			✓	high
3-(heptanoyloxy)−4-(trimethylazaniumyl)butanoate	Fatty Acyls	274.20109 275.20456	M + H M + 2 H	3.03			✓	high
3-{[(5Z)−3-hydroxyoct-5-enoyl]oxy}−4-(trimethylazaniumyl)butanoate	Fatty Acyls	302.19547 303.19892	M + H M + 2 H	4.19			✓	high
3-[(2E)-hex-2-enoyloxy]−4-(trimethylazaniumyl)butanoate	Fatty Acyls	258.17035 259.17379	M + H M + 2 H	5.43			✓	high
2,4,6-octatrienal	Fatty Acyls	123.08059 124.08389	M + H M + 2 H	5.34			✓	high
12-chloro-dodecanoic acid	Fatty Acyls	235.14579 236.14932	M + H M + 2 H	5.90			✓	high
methyl 4-[2-(2-formyl-vinyl)−3-hydroxy-5-oxo-cyclopentyl]-butanoate	Fatty Acyls	255.12251 256.12608	M + H M + 2 H	7.40			✓	high
(5Z,7E)−9,10-seco-5,7,10(19)-cholestatriene	Sterol Lipids	369.35150 370.35492	M + H M + 2 H	10.52	✓		✓	high
26,26,26-trifluoro-25-hydroxy-27-norvitamin D3	Sterol Lipids	441.29831	M + H	10.42	✓			medium
(6RS)−6,19-epidioxy-24,24-difluoro-25-hydroxy-6,19-dihydrovitamin D3	Sterol Lipids	469.31338 470.31429	M + H M + 2 H	6.89			✓	high
cholesta-5,7,8(14),22E-tetraen-3-one	Sterol Lipids	379.30122	M + H	8.42	✓			medium
(25 R)−3α,7α-dihydroxy-5β-cholestan-27-oyl taurine	Sterol Lipids	542.35356	M + H	5.20		✓		medium
1-octadecanoyl-rac-glycerol	Glycerolipids	359.31578	M + H	8.87	✓			medium
1-tetradecanoyl-glycero-3-phospho-(1-sn-glycerol)	Glycerophospholipids	457.25547	M + H	9.00			✓	high
1-(4Z,7Z,10Z,13Z,16Z,19Z-docosahexaenoyl)-glycero-3-phosphoserine	Glycerophospholipids	570.28281 572.28908 571.28463	M + H M + 3 H M + 2 H	6.86			✓	high
1-(1Z-eicosenyl)−2-(4Z,7Z,10Z,13Z,16Z,19Z-docosahexaenoyl)-glycero-3-phosphoserine	Glycerophospholipids	848.57957	M + H	10.47			✓	high
1-(7Z,10Z,13Z,16Z-docosatetraenoyl)-glycero-3-phosphocholine	Glycerophospholipids	572.37175 573.37447	M + H M + 2 H	7.72			✓	high

^*^For this compound, medium confidence match refers to *in vivo* SPME, high confidence match refers to SLE

The annotation was based on intensity profiles, retention time, mass defect, and isotope/adduct patterns of peaks. In high confidence match, non-zero xMSannotator multistage score, required adducts, N, O, P, S/C ratio check, hydrogen/carbon ratio check, abundance ratio checks for isotopes, multimers and multiply charged adducts are satisfied; in medium confidence match, pathway level correlation is satisfied^18^.

In contrast, SLE method used for lipid extraction from homogenized fish muscle samples revealed the presence of many lipid compounds with higher molecular masses (up to 900 *m/z*) (Fig. 1c) not detected in *in vivo* or *ex vivo* SPME extracts (Table 1 and Supplementary Table 1). Owing to the employment of exhaustive extraction conditions along with sonication and the use of organic solvents, SLE enabled the release of high-abundant lipids such as sterols and glycerophospholipids from membrane and intracellular environments, and possibly of lipids belonging to aggregated structures formed during sample storage, which does not represent the unbound fraction of labile lipids detected *in vivo*. However, the lipid-soluble active form of vitamin D_3_ ((5Z,7E)-9,10-seco-5,7,10(19)-cholestatriene) detected in *in vivo* SPME extracts was also found in SLE samples, but not after *ex vivo* SPME, indicating that certain lipids found in their unbound form in living systems may undergo binding reactions as a result of storage. It should be also emphasized that in this study frozen fish muscle samples were not subjected to freeze-thaw cycles during one-year storage period and the observed differences in the lipid profile are not related to the freezing/thawing processes. Homogenization of stored tissue and application of exhaustive sample preparation method (SLE) released compounds from the aggregated structures and facilitated their detection. SLE technique has a distinct advantage in releasing lipids from their complexes with cellular components, such as proteins. Moreover, disintegration of lipid bilayer makes this method highly efficient in the analysis of high-abundant membrane lipids, such as glycerophospholipids, which are not present in the free form in the tissues and cannot be extracted with the use of SPME technique.

However, SPME, as a low-invasive technique, does not disrupt cell membrane structure and facilities the extraction of specific lipids present in unbound form in the system under study. Therefore, low-abundant and short-lived tissue components, such as signaling molecules might be captured *in vivo* by SPME probes, providing the insight into lipidome of the living system. Additionally, quenching of the extracted lipids during *in vivo* SPME approach prevents their oxidative degradation, a process commonly occurring during sample collection and handling with the use of traditional methodologies^20,27^. Also, possible alterations in the free fraction of lipid composition during sample storage resulting from enzymatic and non-enzymatic transformations can be monitored with the use of SPME method.

## Conclusions

To summarize, results obtained from SPME of muscle tissue performed in living fish (*in vivo* SPME) and in muscle samples submitted to a one year storage period (*ex vivo* SPME) were compared and contrasted with results obtained from SLE of homogenized tissue samples, with aims of investigating the real-time profile of biological lipids and its potential disruption during sample preparation and storage. *In vivo* SPME sampling, as a low invasiveness microextraction method, was shown to enable the detection of unstable molecules, such as fatty acyls, known to be present in trace amounts in tissue of living organisms^7^. In addition, application of *ex vivo* SPME provided insight into alterations to the lipid composition of samples possibly stemming from the induction or suppression of chemical and biological processes throughout the storage period that result in the conversion of unbound lipids to a bound state. The findings obtained through comparisons of compounds identified in *in vivo* SPME extracts versus *ex vivo* SPME and SLE extracts sampled following a year of storage support the assertion that factors such as sample handling and storage have a profound effect on the detection of biological lipids and thus the characterization of the lipidome profile of a given system.

## Materials and Methods

### Materials

LC-MS grade acetonitrile (ACN), methanol, and water were purchased from Fisher Scientific (Ottawa, ON, Canada). Hexane (Hex) and acetone (Ace) were purchased from Sigma-Aldrich (Oakville, ON, Canada). Biocompatible SPME mixed mode probes (45 µm thickness, 15 mm length of coating) were provided by Supelco (Bellefonte, PA, USA). Standards used for preparation of instrumental QC samples (trans-4-(Aminomethyl)cyclohexanecarboxylic acid, Tranexemic acid, Phenylalanine-d5, Phenylalanine, Tryptophan, Progesterone) were purchased from Sigma-Aldrich (Oakville, ON, Canada).

### *In vivo* SPME sampling

Juvenile rainbow trout (*Oncorhynchus mykiss*) used in this study (13.6 ± 0.8 cm, 24.9 ± 3.8 g, n = 5) were purchased from Silver Creek Aquaculture (Erin, ON Canada). Fish were acclimatized to laboratory conditions in continuously flowing non-chlorinated water, and fed 2.0 Pt floating commercial trout ration (Martin’s Feed Mill, ON Canada). All fibers used in *in vivo* investigations were preconditioned in methanol/water (50/50, v/v) prior to analysis. *In vivo* sampling of fish muscle tissue was conducted by inserting mixed mode SPME fibers into the dorsal-epaxial muscle (near the dorsal fin) of fish initially immobilized with the use of a large foam bed. After insertion of fibers, fish were held in an aerated, covered bucket for a 20 minute period while extractions were carried out. Following extraction and the subsequent removal of fibers from the tissue, all fibers were rinsed with nanopure water to remove any excess matrix components adhered to the coating, and immersed for 90 min in 300 µL of acetonitrile/water (80/20, v/v) for desorption of lipids from the SPME coatings, with vortex agitation set at 1,000 rpm. Extract solutions were then injected to the HPLC-ESI-MS system for instrumental analysis. After completion of *in vivo* tissue sampling, fish were anaesthetized with 0.1% ethyl 3-amino benzoate methanesulfonate, and killed by spinal severance. Fish muscle samples were collected and immediately frozen in liquid nitrogen for further SPME sampling. All experimental protocols were in accordance with and approved by the University of Waterloo Animal Care Committee (AUPP # 14–16).

### *Ex vivo* SPME sampling

After undergoing storage at −80 °C for one year, fish muscle tissues were submitted to analysis by *ex vivo* SPME. Prior to sampling, all employed mixed mode fibers were preconditioned in methanol/water (50/50, v/v). *Ex vivo* sampling was performed by inserting fibers into non-homogenized muscle tissue for a 20 min period. Following extraction, fibers were removed from tissue, rinsed with nanopure water, and immersed for 90 min in 300 µL of acetonitrile/water (80/20, v/v) for desorption of lipids from SPME coatings, using vortex agitation at 1,000 rpm. The obtained extract solutions were then injected to the HPLC-ESI-MS system for instrumental analysis.

### Solid-liquid extraction (SLE)

Solid-liquid extractions were carried out according to the modified Mijangos method^28^. In brief, after performing *ex vivo* SPME analysis on non-homogenized tissue, 0.5 g of fish muscle was homogenized in dry ice, then placed in a tube. Following, 10 mL of Hex:Ace (50:50, v/v) was added to the sample, which was then submitted to ultrasonic assisted solid-liquid extraction (Bransonic® CPX2800H, Branson) for a 15 min period. The obtained mixture was then allowed to settle for 15 min, after which 7 mL of supernatant was collected and transferred to a new tube. The same outlined procedure was then repeated twice. All collected supernatant (21 mL) was evaporated to dryness under a nitrogen gas stream, and reconstituted in 2 mL of desorption solution used in SPME (acetonitrile/water (80/20, v/v)). The obtained extracts were then centrifuged for 5 min at 4000 rpm in tubes equipped with a 0.65 μm pore DVPP filter (Ultrafree-CL DV Centrifugal Filter, Millipore Sigma) prior to LC/MS analysis.

### Liquid chromatography/mass spectrometry analysis (LC/MS)

Metabolite profiling was accomplished with the use of an LC/MS system consisted of a ThermoAccela autosampler, pumps, and an Exactive Benchtop Orbitrap System (Thermo Fisher Scientific, CA, USA). Metabolites were separated in reversed-phase, using a pentafluorophenyl column (Kinetex Phenomenex, 2.1 mm × 100 mm, 1.7 µm particle size). The flow rate was set at 300 µL/min. Mobile phase A consisted of water/formic acid (99.9/0.1, v/v), while mobile phase B consisted of acetonitrile/formic acid (99.9/0.1, v/v). The starting mobile phase conditions were 90% A from 0 to 1.0 min, followed by a linear gradient to 10% A from 1.0 to 9.0 min, and an isocratic hold at 10% A until 12.0 min. Total run time was 18 min per sample, including a 6-min re-equilibration period. Injection volume was 10 µL. Analyses were performed in positive ionization mode with a mass range of m/z 100–1000. To maintain a mass accuracy better than 5 ppm, the Exactive Benchtop Orbitrap was calibrated with a MSCAL5 standard solution (caffeine, tetrapeptide “Met-Arg-Phe-Ala”, ultramark 1621). In order to keep the instrumental conditions constant, the robustness of LC-MS method was verified via the calibration of the instrument every 24 hours during the analysis and also by using instrumental quality control samples as well as blank samples. Additionally, the developed analytical method was validated by the principal component analysis (PCA) (Supplementary Fig. 1). Score Plot showed that the pooled QCs present a stable trend within *in vivo* SPME and *ex vivo* SPME sampling. SPME fibers presented a stable performance within the extracted peaks during *in vivo* and *ex vivo* experiments (Supplementary Fig. 2).

### Data processing

Raw LC/MS data sets were processed using XCMS package software for peak extraction, grouping, retention time correction, and peak filling^29–31^. All parameters for XCMS analysis were optimized by IPO package^32^. Extracted peaks were annotated with the use of the xMSannotator Integrative Scoring Algorithm^19^. LIPID MAPS was employed as a reference database. The annotated molecules are tentative identification, where unique features with medium to high confidence matches annotated by LIPID MAPS were selected for further discussion. The entire data processing procedure was wrapped into R-script^33^, which can be found in a separate section.

### R-script for data analysis

# loading the package

library(xcms)

library(BiocParallel)

library(IPO)

library(xMSannotator)

# the following code for XCMS optimation with IPO package

mzdatapath <- “data/positive/pqc/“

mzdatafiles <- list.files(mzdatapath, recursive = TRUE, full.names = TRUE)

peakpickingParameters <- getDefaultXcmsSetStartingParams(‘centWave’)

#setting levels for min_peakwidth to 10 and 20 (hence 15 is the center point)

peakpickingParameters$min_peakwidth <- c(2,10)

peakpickingParameters$max_peakwidth <- c(15,25)

#setting only one value for ppm therefore this parameter is not optimized

peakpickingParameters$ppm <- 2.5

resultPeakpicking <-

 optimizeXcmsSet(files = mzdatafiles[1:4],

  params = peakpickingParameters,

  nSlaves = 12,

  subdir = ‘rsm’)

optimizedXcmsSetObject <- resultPeakpicking$best_settings$xset

retcorGroupParameters <- getDefaultRetGroupStartingParams()

retcorGroupParameters$profStep <- 1

resultRetcorGroup <-

 optimizeRetGroup(xset = optimizedXcmsSetObject,

  params = retcorGroupParameters,

  nSlaves = 12,

  subdir = “results”)

writeRScript(resultPeakpicking$best_settings$parameters,

  resultRetcorGroup$best_settings,

  nSlaves = 12)

# the following code is combination the data process into one function

getopqedata <- function(path,

  index = F,

  xsmethod = “centWave”,

  peakwidth = c(14, 25),

  ppm = 2.5,

  noise = 0,

  snthresh = 10,

  mzdiff = −0.00395,

  prefilter = c(3, 100),

  mzCenterFun = “wMean”,

  integrate = 1,

  fitgauss = FALSE,

  verbose.columns = FALSE,

  BPPARAM = BiocParallel::SnowParam(workers = 12),

  rmethod = “obiwarp”,

  plottype = “none”,

  distFunc = “cor_opt”,

  profStep = 1,

  center = 2,

  response = 1,

  gapInit = 0.6176,

  gapExtend = 2.4,

  factorDiag = 2,

  factorGap = 1,

  localAlignment = 0,

  gmethod = “density”,

  bw = 0.25,

  mzwid = 0.0021748,

  minfrac = 1,

  minsamp = 1,

  gmax = 50,

  …) {

 cdffiles <- list.files(path, recursive = TRUE, full.names = TRUE)

 if (index) {

  cdffiles <- cdffiles[index]

 }

 xset <- xcms::xcmsSet(

  cdffiles,

  method = xsmethod,

  snthresh = snthresh,

  mzdiff = mzdiff,

  BPPARAM = BPPARAM,

  peakwidth = peakwidth,

  ppm = ppm,

  noise = noise,

  prefilter = prefilter,

  mzCenterFun = mzCenterFun,

  integrate = integrate,

  fitgauss = fitgauss,

  verbose.columns = verbose.columns,

  …

 )

 if (index & length(index) == 1) {

  xset3 <- xset

 } else{

  xset <- xcms::group(

  xset,

  method = gmethod,

  bw = bw,

  mzwid = mzwid,

  minfrac = minfrac,

  minsamp = minsamp,

  max = gmax

 )

 xset2 <- xcms::retcor(

  xset,

  method = rmethod,

  plottype = plottype,

  distFunc = distFunc,

  profStep = profStep,

  center = center,

  response = response,

  gapInit = gapInit,

  gapExtend = gapExtend,

  factorDiag = factorDiag,

  factorGap = factorGap,

  localAlignment = localAlignment

 )

 # you need group the peaks again for this corrected data

 xset2 <- xcms::group(

  xset2,

  method = gmethod,

  bw = bw,

  mzwid = mzwid,

  minfrac = minfrac,

  minsamp = minsamp,

  max = gmax

 )

 xset3 <- xcms::fillPeaks(xset2, BPPARAM = BPPARAM)

}

 return(xset3)

}

# the following code is used to process the data

path <- “./data/lipid/exvivo/“

xset <- getopqedata(path)

path <- “./data/lipid/invivo/“

xset2 <- getopqedata(path)

path <- “./data/lipid/le/“

xset3 <- getopqedata(path)

# get the peaks group infomation for histgram

xd1 <- as.data.frame(xset@groups)

xd2 <- as.data.frame(xset2@groups)

xd3 <- as.data.frame(xset3@groups)

write.csv(xd1,‘exvivo.csv’)

write.csv(xd2,‘invivo.csv’)

write.csv(xd3,‘le.csv’)

# make annotation with one function from xMSannotator package

fanno <-

 function(xset,

  outloc = “./result/“,

  mode = ‘pos’,

  list = c(

   “M + 2 H”,

   “M + H + NH4”,

   “M + ACN + 2 H”,

   “M + 2ACN + 2 H”,

   “M + H”,

   “M + NH4”,

   “M + Na”,

   “M + ACN + H”,

   “M + ACN + Na”,

   “M + 2ACN + H”,

   “2 M + H”,

   “2 M + Na”,

   “2 M + ACN + H”,

   “M + 2Na-H”,

   “M + H-H2O”,

   “M + H-2H2O”

 ),

 db_name = ‘HMDB’, …) {

 data <- xcms::groupval(xset, ‘medret’, “into”)

 adduct_weights = cbind.data.frame(Adduct = c(‘M + H’,‘M-H’),Weight = c(5,5))

 mz <- xcms::groups(xset)[, 1]

 time <- xcms::groups(xset)[, 4]

 data <- as.data.frame(cbind(mz, time, data))

 data <- unique(data)

 if (mode =  = ‘neg’) {

 annotres <-

 xMSannotator::multilevelannotation(

   dataA = data,

   max.mz.diff = 5,

   max.rt.diff = 10,

   cormethod = “pearson”,

   queryadductlist = list,

   mode = mode,

   outloc = outloc,

   db_name = db_name,

   adduct_weights = adduct_weights,

   num_sets = 1000,

   allsteps = TRUE,

   corthresh = 0.7,

   NOPS_check = TRUE,

   customIDs = NA,

   missing.value = NA,

   hclustmethod = “complete”,

   deepsplit = 2,

   networktype = “unsigned”,

   minclustsize = 10,

   module.merge.dissimilarity = 0.2,

   filter.by = c(“M-H”),

   biofluid.location = NA,

   origin = NA,

   status = “Detected and Quantified”,

   boostIDs = NA,

   max_isp = 5,

   HMDBselect = “union”,

   mass_defect_window = 0.01,

   pathwaycheckmode = “pm”,

   mass_defect_mode = mode

  )

 }else{

  annotres <-

  xMSannotator::multilevelannotation(

   dataA = data,

   max.mz.diff = 5,

   max.rt.diff = 10,

   cormethod = “pearson”,

   queryadductlist = list,

   mode = mode,

   outloc = outloc,

   db_name = db_name,

   adduct_weights = adduct_weights,

   num_sets = 1000,

   allsteps = TRUE,

   corthresh = 0.7,

   NOPS_check = TRUE,

   customIDs = NA,

   missing.value = NA,

   hclustmethod = “complete”,

   deepsplit = 2,

   networktype = “unsigned”,

   minclustsize = 10,

   module.merge.dissimilarity = 0.2,

   filter.by = c(“M + H”),

   biofluid.location = NA,

   origin = NA,

   status = “Detected and Quantified”,

   boostIDs = NA,

   max_isp = 5,

   HMDBselect = “union”,

   mass_defect_window = 0.01,

   pathwaycheckmode = “pm”,

   mass_defect_mode = mode

   )

  }

  return(annotres)

 }

# annotate the data

annoexvivo <- fanno(xset,outloc = ‘data/lipid/exvivo/sub/‘,db_name = ‘LipidMaps’,num_nodes = 12)

annoinvivo <- fanno(xset2,outloc = ‘data/lipid/invivo/sub/‘,db_name = ‘LipidMaps’,num_nodes = 12)

anole <- fanno(xset3,outloc = ‘data/lipid/le/sub/‘,db_name = ‘LipidMaps’,num_nodes = 12).

## Electronic supplementary material

Supplementary Information
