# Supplementary material for: Tissue storage affects lipidome profiling in comparison to in vivo microsampling approach
Source: Sci Rep. 2018 May 3;8:6980. doi: 10.1038/s41598-018-25428-2 (PMC5934459; doi:10.1038/s41598-018-25428-2)
Supplement: Supplementary file 1 — Supplementary Information [file 41598_2018_25428_MOESM1_ESM.docx]

**Tissue storage affects lipidome profiling in comparison to *in vivo* microsampling approach**

Anna Roszkowska^1*^, Miao Yu^1^, Vincent Bessonneau^1^, Leslie Bragg^2^, Mark Servos^2^, Janusz Pawliszyn^1#^

^1^Department of Chemistry, University of Waterloo, ON, Canada

^2^Department of Biology, University of Waterloo, ON, Canada

^*^Current address: Department of Pharmaceutical Chemistry, Medical University of Gdańsk, Gdańsk, Poland

^#^Corresponding author; e-mail address: janusz@uwaterloo.ca (J. Pawliszyn); phone: +1-519-

888-4567 ext: 35123; fax: +1-519-888-4348

**
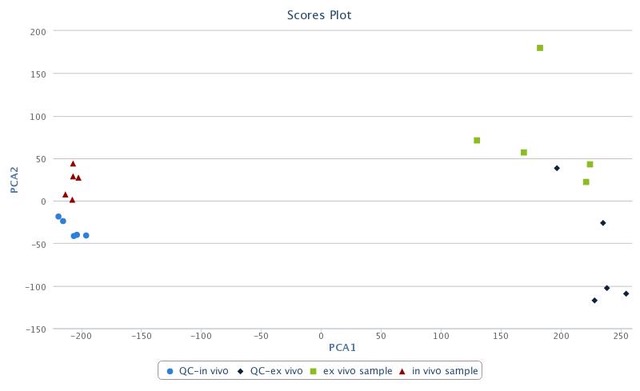
**

**Supplementary Fig. 1.** PCA plot presenting clusters of data obtained for *in vivo* SPME and *ex vivo* SPME sampling, and for QC prepared from pooled aliquots of analyzed samples used for additional monitoring of instrument performance.

**Supplementary Fig. 2.** The stability of performance of the methods applied in the study. The preliminary experiments (triplicates sampling of fish muscle for the same fish) were performed in order to present the stability of *in vivo* SPME, *ex vivo* SPME and SLE. The median %RSD of all peaks from *in vivo* SPME (1459 detected peaks), *ex vivo* SPME (918 detected peaks) and SLE (2480 detected peaks) were 34.83%, 64.93% and 75.99%, respectively.

**Table S1 Unique features annotated with medium confidence matches in extracts obtained by solid-liquid extraction by LIPID MAPS.**

| **Compound** | **Category** | **Precursor m/z** | **Precursor adducts** | **RT (min)** |
| --- | --- | --- | --- | --- |
| pentanamide | Fatty Acyls | 102.09155 | M+H | 1.20 |
| N-methyl hexanoyl amine | Fatty Acyls | 130.12262 | M+H | 2.10 |
| 2,5-Diaminopentanoic acid | Fatty Acyls | 133.09706 | M+H | 1.19 |
| 3-oxo-5S-amino-hexanoic acid | Fatty Acyls | 146.08123 | M+H | 0.88 |
| 3,4,5-trihydroxy-hexanoic acid | Fatty Acyls | 165.07604 | M+H | 6.58 |
| 3,7-dimethyloctane-1,7-diol | Fatty Acyls | 175.16897 | M+H | 5.70 |
| 10-hydroxy-8E-Decene-2,4,6-triynoic acid | Fatty Acyls | 177.05458 | M+H | 6.75 |
| 7,8-diaminononanoic acid | Fatty Acyls | 189.15995 | M+H | 1.33 |
| 3,5,7,9,11-dodecapentaenoic acid | Fatty Acyls | 191.10715 | M+H | 6.02 |
| 6,10-Dimethyl-5(E)9-undecadien-2-one | Fatty Acyls | 195.17419 | M+H | 7.63 |
| dodecanamide | Fatty Acyls | 200.20111 | M+H | 9.15 |
| 12-oxo-5E,8E,10Z-dodecatrienoic acid | Fatty Acyls | 209.11647 | M+H | 5.37 |
| 4-(5-aminopentyl)(hydroxy) amino-4-oxobutanoic acid | Fatty Acyls | 219.13410 | M+H | 0.95 |
| 5,7,9,11,13-tetradecapentaenoic acid | Fatty Acyls | 219.13858 | M+H | 8.88 |
| 2E,4E,8E,10E-Dodecatetraenedioic acid | Fatty Acyls | 223.09689 | M+H | 6.05 |
| 6-(6-aminohexanamido) hexanoic acid | Fatty Acyls | 245.18642 | M+H | 2.35 |
| 9Z-hexadecenamide | Fatty Acyls | 254.24861 | M+H | 8.11 |
| 3-O-acetyl-1-deoxy-1-(4-methoxyphenyl) pentitol | Fatty Acyls | 285.13379 | M+H | 3.41 |
| 3-(2E)-5-carboxypent-2-enoyloxy-4-(trimethylazaniumyl) butanoate | Fatty Acyls | 288.14405 | M+H | 6.49 |
| 3-(6-oxodecanoyl)oxy-4-(trimethylazaniumyl) butanoate | Fatty Acyls | 330.22675 | M+H | 9.34 |
| 3-(9Z)-3-hydroxydodec-9-enoyloxy-4-(trimethylazaniumyl)butanoate | Fatty Acyls | 358.25915 | M+H | 7.05 |
| 3-(icosanoyloxy)-4-(trimethylazaniumyl) butanoate | Fatty Acyls | 456.40372 | M+H | 12.82 |
| (2E,4E)-N-(2-methylpropyl)octadeca-24-dienamide | Fatty Acyls | 336.32700 | M+H | 9.25 |
| N-(9Z-octadecenoyl)-glycine | Fatty Acyls | 340.28464 | M+H | 9.21 |
| N-hexadecanoyl-glutamic acid | Fatty Acyls | 386.29090 | M+H | 7.05 |
| N-hexadecanoyl-phenylalanine | Fatty Acyls | 404.31617 | M+H | 9.98 |
| N-(9Z-octadecenoyl)-phenylalanine | Fatty Acyls | 430.33099 | M+H | 10.06 |
| N-(9Z-octadecenoyl)-methionine | Fatty Acyls | 414.30459 | M+H | 8.89 |
| N-(5Z,8Z,11Z,14Z,17Z-eicosapentaenoyl)-ethanolamine | Fatty Acyls | 346.27351 | M+H | 7.77 |
| 12,15-epoxy-13,14-dimethyl-12,14-eicosadienoic acid | Fatty Acyls | 351.28999 | M+H | 9.35 |
| 1,10Z-heptadecadiene-2,3R-dicarboxylic acid | Fatty Acyls | 353.26852 | M+H | 8.04 |
| 15-oxo-18Z-tetracosenoic acid | Fatty Acyls | 381.33664 | M+H | 9.40 |
| (5R,6Z,8E,10E,14Z)-5,20,20,20-tetrahydroxy-12-oxoicosa-6,8,10,14-tetraenoic acid | Fatty Acyls | 383.20516 | M+H | 8.21 |
| (5S,6R,7E,9E,12S,14Z)-6-{[(2S)-2-amino-2-carboxyethyl]sulfanyl}-5,12-  dihydroxyicosa-7,9,14-trienoic acid | Fatty Acyls | 458.25751 | M+H | 9.00 |
| 1-eicosanoyl-rac-glycerol | Glycerolipids | 387.34710 | M+H | 9.30 |
| 1-(9Z,12Z,15Z-octadecatrienoyl)-2-hexadecanoyl-3-O-alpha-D-glucuronosyl-sn-glycerol | Glycerolipids | 767.53072 | M+H | 10.16 |
| 1-(9Z,12Z-heptadecadienoyl)-glycero-3-phosphate | Glycerophospholipids | 421.23331 | M+H | 7.78 |
| 1-(1Z-hexadecenyl)-sn-glycero-3-phosphoethanolamine | Glycerophospholipids | 438.29780 | M+H | 7.43 |
| 1-hexadecanoyl-sn-glycero-3-phosphoserine | Glycerophospholipids | 498.28321 | M+H | 7.00 |
| 1-(10Z,13Z,16Z-nonadecatrienoyl)-sn-glycero-3-phosphocholine | Glycerophospholipids | 532.34076 | M+H | 7.76 |
| 1-(5Z,8Z,11Z,14Z-eicosatetraenoyl)-sn-glycero-3-phosphoserine | Glycerophospholipids | 546.28379 | M+H | 6.82 |
| 1-(4Z,7Z,10Z,13Z,16Z,19Z-docosahexaenoyl)-glycero-3-phospho-(1-sn-glycerol) | Glycerophospholipids | 557.28816 | M+H | 7.03 |
| 1-(11Z-docosenoyl)-glycero-3-phosphoserine | Glycerophospholipids | 580.35998 | M+H | 7.75 |
| 1-octadecyl-glycero-3-phospho-(1-myo-inositol) | Glycerophospholipids | 587.35472 | M+H | 7.42 |
| 1-eicosyl-glycero-3-phospho-(1-myo-inositol) | Glycerophospholipids | 615.38495 | M+H | 7.85 |
| 1-hexadecyl-2-tridecanoyl-glycero-3-phosphoethanolamine | Glycerophospholipids | 636.49390 | M+H | 9.25 |
| 1-(1Z-hexadecenyl)-2-dodecanoyl-glycero-3-phosphoserine | Glycerophospholipids | 664.45699 | M+H | 12.13 |
| 1-hexadecyl-2-tridecanoyl-glycero-3-phospho-(1-sn-glycerol) | Glycerophospholipids | 667.48906 | M+H | 9.36 |
| 1-hexadecyl-2-tridecanoyl-glycero-3-phosphoserine | Glycerophospholipids | 680.48428 | M+H | 7.81 |
| 1-O-(1Z-hexadecenyl)-2-(4Z,7Z,10Z,13Z,16Z,19Z-docosahexaenoyl)-sn-glycero-3-phosphoethanolamine | Glycerophospholipids | 748.52424 | M+H | 10.33 |
| 1-(8-5-ladderane-octanoyl)-2-(8-3-ladderane-octanyl)-sn-glycerophosphoethanolamine | Glycerophospholipids | 772.52660 | M+H | 10.08 |
| 1-(1Z-octadecenyl)-2-(4Z,7Z,10Z,13Z,16Z,19Z-docosahexaenoyl)-sn-glycero-3-phosphoethanolamine | Glycerophospholipids | 776.56143 | M+H | 10.49 |
| 1-(6-3-ladderane-hexanoyl)-2-(8-3-ladderane-octanyl)-sn-glycerophosphocholine | Glycerophospholipids | 788.56051 | M+H | 10.77 |
| 1-eicosyl-2-(4Z,7Z,10Z,13Z,16Z,19Z-docosahexaenoyl)-glycero-3-phosphoethanolamine | Glycerophospholipids | 806.60286 | M+H | 12.55 |
| 1-(8-5-ladderane-octanoyl)-2-(8-3-ladderane-octanyl)-sn-glycerophosphocholine | Glycerophospholipids | 814.57689 | M+H | 10.69 |
| 1-(1Z-octadecenyl)-2-(4Z,7Z,10Z,13Z,16Z,19Z-docosahexaenoyl)-sn-glycero-3-phosphocholine | Glycerophospholipids | 818.60811 | M+H | 11.98 |
| 1-(1Z-eicosenyl)-2-heneicosanoyl-glycero-3-phosphoserine | Glycerophospholipids | 846.65472 | M+H | 8.36 |
| 1-eicosyl-2-(4Z,7Z,10Z,13Z,16Z,19Z-docosahexaenoyl)-glycero-3-phosphoserine | Glycerophospholipids | 850.59811 | M+H | 10.69 |
| 12-di-(4Z,7Z,10Z,13Z,16Z,19Z-docosahexaenoyl)-sn-glycero-3-phosphocholine | Glycerophospholipids | 878.56526 | M+H | 10.33 |
| 1-(1Z-eicosenyl)-2-(9Z-nonadecenoyl)-glycero-3-phospho-(1-myo-inositol) | Glycerophospholipids | 891.62933 | M+H | 11.19 |
| 1-(1Z-eicosenyl)-2-(4Z,7Z,10Z,13Z,16Z,19Z-docosahexaenoyl)-sn-glycero-3-phospho-(1-myo-inositol) | Glycerophospholipids | 923.59857 | M+H | 10.23 |
| (4S)-4-hydroxy-3,5,5-trimethyl-4-(1E)-3-oxobut-1-en-1-ylcyclohex-2-en-1-one | Prenol Lipids | 223.13270 | M+H | 6.30 |
| 8,8-Diapocarotene-8,8-dioic acid | Prenol Lipids | 329.17466 | M+H | 6.65 |
| 2-methyl-3-((2E,10E,14E,18E,22E,26E)-3,7,11,15,19,23,27,31-  octamethyldotriaconta-2,10,14,18,22,26,30-heptaenyl) naphthalene-1,4-dione | Prenol Lipids | 719.57732 | M+H | 13.13 |
| Hexadecasphinganine | Sphingolipids | 274.27443 | M+H | 13.99 |
| Sphinganine-1-phosphocholine | Sphingolipids | 467.35862 | M+H | 7.26 |
| N-(2-hydroxy-eicosanoyl)-pentadecasphing-4-enine-1-phosphoethanolamine | Sphingolipids | 691.53781 | M+H | 9.16 |
| N-(15Z-tetracosenoyl)-sphing-4-enine | Sphingolipids | 648.62910 | M+H | 11.75 |
| N-(2-hydroxy-eicosanoyl)-4E,6E-pentadecasphingadienine-1-phosphoethanolamine | Sphingolipids | 689.52347 | M+H | 7.67 |
| N-(tetradecanoyl)-1-b-lactosyl-sphing-4-enine | Sphingolipids | 834.59169 | M+H | 11.19 |
| N-docosanoyl-1-beta-(3-sulfo)-glucosyl-sphing-4-enine | Sphingolipids | 864.62535 | M+H | 7.57 |
| 17beta-hydroxy-5alpha-1-oxo-23-seco-androstan-3-oic acid | Sterol Lipids | 323.22240 | M+H | 7.06 |
| (1S,3R,5Z,7E)-1,3-dihydroxy-2,4-nor-9,10-secochola-5,7,10(19)-trien-2,3-al | Sterol Lipids | 359.25748 | M+H | 6.86 |
| (5Z,7E)-(1S,3R)-24-nor-9,10-seco-5,7,10(19)-cholatriene-1,3,23-triol | Sterol Lipids | 361.27364 | M+H | 5.89 |
| (5Z,7E)-(3S)-26,26,26-trifluoro-27-nor-9,10-seco-5,7,10(19)-cholestatriene-3,25-diol | Sterol Lipids | 441.29872 | M+H | 9.95 |
| 28-nor-3-oxo-18-acetoxy-with-4-enolide | Sterol Lipids | 457.29498 | M+H | 9.60 |
| (7E)-(1R3R)-2424-difluoro-19-nor-910-seco-57-cholestadiene-132526-tetrol | Sterol Lipids | 457.31449 | M+H | 7.94 |
| (22R)-2beta3beta1422-tetrahydroxy-6-oxo-5beta-cholest-7-en-25-yl D-glucopyranoside | Sterol Lipids | 627.37691 | M+H | 7.56 |
| 3-Oxochola-146-trien-24-oic Acid | Sterol Lipids | 369.24203 | M+H | 9.77 |
| 3-O-(6-O-(7Z10Z13Z-hexadecatrienoyl)-beta-D-glucopyranosyl)-cholest-5-en-3beta-ol | Sterol Lipids | 781.59451 | M+H | 9.73 |
| 3-O-(6-O-(7Z10Z13Z-hexadecatrienoyl)-beta-D-glucopyranosyl)-campest-5-en-3beta-ol | Sterol Lipids | 795.61195 | M+H | 12.75 |
| N-3alpha-(sulfooxy)-5beta-cholan-24-oylglycine | Sterol Lipids | 514.28428 | M+H | 4.55 |
| cholest-5-en-3beta-yl beta-D-glucopyranoside | Sterol Lipids | 549.41540 | M+H | 7.70 |
| (5Z,7E)-(1S,3R)-24,24-difluoro-26,27-dimethyl-9,10-seco-5,7,10(19)-cholestatrien-1,3,25-triol | Sterol Lipids | 481.34742 | M+H | 7.74 |
